# Impact of Hydrophobic Pollutants' Behavior on Occupational and Environmental Health

**DOI:** 10.1100/tsw.2005.28

**Published:** 2005-03-19

**Authors:** Ijeoma Kanu, Ebere Anyanwu

**Affiliations:** ^1^ Department of Microbiology, Abia State University, Uturu, Abia State, Nigeria; ^2^ Medical Center for Immune and Toxic Disorders, 25010 Oakhurst Drive, Suite 200, Spring, TX, USA

**Keywords:** hydrophobic pollutants, biological uptake, bioaccumulation, bioconcentration, biomagnifications, photochemical, microbial degradation, environmental hazard and risk, cancer

## Abstract

This paper reviews the influence of hydrophobic pollutant behavior on environmental hazards and risks. The definition and examples of hydrophobic pollutants are given as a guide to better understand the sources of release and the media of dispersion in the environment. The properties and behavior of hydrophobic pollutants are described and their influence on environmental hazard and risk is reviewed and evaluated. The overall outcome of the assessment and evaluation showed that all hydrophobic pollutants are hazardous and risky to all organisms, including man. Their risk effects are due to their inherent persistence, bioaccumulation potential, environmental mobility, and reactivity. Their hazardous effects on organisms occur at varying spatial and temporal degrees of emissions, toxicities, exposures, and concentrations.

## INTRODUCTION

Advances in technology, industrial development, and human activities have led to the increased use of chemicals, most of which are interfering with human health and the normal balance of ecological systems. It is on these grounds that environmental scientists and governments have sought — and are still seeking — a clear definition of what constitutes environmental pollution and the effects on ecosystems. Hence, the need to monitor, control, and remedy the release of chemical pollutants into the environment has become a serious public and environmental health issue. The Environmental Protection Act (EPA)[1] refers to "pollution of the environment as due to the release into the environmental media (air, water and soil) from any processes or substances, which are capable of causing harm to man or any other living organism supported by the environment." Broadly speaking, all chemicals are potential pollutants depending on the concentration, interaction with the environment, and the effects on living organisms and ecosystems. The only possible way to minimize the environmental burden they cause is to strictly limit or prevent the release. This paper focuses on "hydrophobic" pollutants and the influence of their behavior on human, occupational, and environmental health.

## HYDROPHOBIC POLLUTANTS

Hydrophobic pollutants are those nonpolar molecules that are either deliberately discharged into the environment through occupational and commercial operations or by inevitable circumstances involving operational accidents, thereby creating environmental health hazards and risks. These include all hydrocarbons, especially industrial chemicals such as polycyclic aromatic hydrocarbons (PAHs) and their derivatives, polychlorinated biphenyls (PCBs), and organic pesticides such as dioxins, chlorinated dibenzofurans, and their derivatives. PAHs belong to a large chemical family comprising many different compounds with important biological activity in mutagenic and carcinogenic processes. They are formed during the thermal degradation of any material composed of carbon and hydrogen. As a consequence of their origin and formation processes, PAHs can be found as constituents of smoke from engines, incineration plants, central heating, and cigarettes[2], while the major source of PCB contamination is scrapping of transformers[3].

### Properties of Hydrophobic Pollutants

Hydrophobic pollutants have a symmetrical distribution of similar atoms to which equal dipoles cancel each other exactly. They all are toxic, persistent, and some are recalcitrant. Their persistence is due to their long environmental half-lives or residence times that take at least 2 years for 95% to degrade[4]. Although those that are recalcitrant are relatively inert and resistant to chemical and biochemical degradation, they tend to be environmentally persistent. They are mobile to some extent and will contaminate environments remote from the release site. All hydrophobic pollutants undergo physical, chemical, and biological processes at varying degrees. For example, they undergo solubility in lipids, phase partitioning, and octanol-water partition coefficient process; volatility and evaporation, sorption and desorption; biological uptake, bioaccumulation, bioconcentration, biomagnifications; chemical and photochemical degradation, and microbial degradation processes. They are very hydrophobic and lipophilic, although a few are moderately less hydrophobic, e.g., phenols, cresols, etc. Their hydrophobicity increases with increasing molecular volume. They are the components of fossil fuels (especially oils, natural gas). Some are derived from partial combustion of any organic matter, for example, PAH. They are generally flammable and have both short- and long-term toxic effects such as narcosis and cancer.

### The Release of Hydrophobic Pollutants in the Environment

Hydrophobic pollutants are released into the environment by natural and anthropogenic emission processes. The natural emission processes include natural fires and natural cycles (such as algal blooms), while the anthropogenic processes include raw material extraction, manufacturing processes, processing, storage (raw materials and products), transport, accidents, product use, and waste disposal processes[5]. The ways by which hydrophobic pollutants are dispersed into the environment involve certain common characteristic pollution events, namely: (1) the pollutant, (2) the source of the pollutant, (3) the transport medium (air, water, or soil), and (4) the target (the organisms, ecosystems, or items of property affected by pollutant). However, there are classical physicochemical principles that explain the means by which the effects of their behavior could be understood better. These include the rate of emission of the pollutants from the source, the rate of transport, chemical and physical transformations they undergo either during transport or after deposition at the target, amounts reaching the target, movement within the target to sensitive organs, and quantification of effects on the target[6,7,8,9].

## Behavior in the Media

In the soil, hydrophobic pollutants are normally adsorbed on soil humic materials. Since most organic matter is found in the surface horizon, there is a tendency for these pollutants to be concentrated in the topsoil. Migration of hydrophobic pollutants down the profile only occurs to any marked extent in highly permeable sandy or graveled soils with low hydrophobic molecular contents and where large pores (macropores) and fissures are present. Organic matter in soils includes decomposing plant material and humic compounds, which have been synthesized by the action of microorganisms on residues of plant material. The humic substances/clay minerals/hydrous oxides are bonded together in various ways and jointly form the colloidal adsorption complex, which plays a very important role in determining the behavior of hydrophobic pollutants[10]. The behavior of hydrophobic pollutants is therefore determined by the interacting processes of oxidation, reduction, adsorption, and precipitation and desorption.

### *Mechanism of Adsorption/Desorption Processes*

When pollutants reach the soil surface, they are either adsorbed with varying strengths on the colloids at the surface of the topsoil or are washed down through the surface layer into the soil profile via rainwater or snow melt. Soluble pollutants will infiltrate into the topsoil in the system of pores where the adsorption of ions occurs. Hydrophobic pollutants will bind to sites on soil organic matter at the soil surface. While in the soil surface, some of the hydrophobic pollutants will undergo photolytic decomposition as a result of exposure to UV wavelengths in daylight. Several different types of adsorption reaction can occur on the surfaces of the hydrophobic pollutants. The extent to which the reactions occur is determined by the composition of the soil, the pH, redox status, and the nature of the contaminants. The more strongly the pollutants are adsorbed, the less likely they are to be leached down the soil profile or to be available for uptake by plants[9]. Furthermore, some hydrophobic pollutants tend to be relatively easily leached in regions where there is a marked excess of precipitation relative to evapotranspiration. In many cases, adsorption is a necessary preliminary stage in the decomposition of hydrophobic pollutant molecules by microbial extracellular enzymes[11]. In addition to the adsorption/desorption processes occurring in the soils, the wide range of microorganisms present also have important effects on the behavior of hydrophobic pollutant molecules. For example, microorganisms such as *Thiobacillus* species catalyze the oxidation of sulfides. It must be emphasized that the sorption characteristics of hydrophobic contaminants depend strongly on the aromaticity of the organic matter in the soil. Therefore, the use of only macroscopic sorbent properties, such as aromaticity, to predict and rationalize sorption values cannot solely be used to explain the behavior of hydrophobic organic contaminants in soil environments[12,13].

### *Enhancing the Sorptive Capabilities of Soil Prior to Cleanup*

Modification of soils with hydrophobic cationic surfactants is an effective approach for enhancing the sorptive capabilities of soil for the purpose of retaining hydrophobic organic contaminants prior to cleanup. This can be done by replacing the cations of loess soil with a cationic surfactant-hexadecyltrimethylammonium (HDTMA) bromide[14]. This is possible because aromatic anions in loess soil are greatly enhanced by modification with HDTMA. The increase of ionic strength and the addition of divalent heavy metal cation Zn (2+) also significantly increases the sorption of aromatic anions on the HDTMA-modified loess soil. In binary solute systems, the sorbed amounts on the HDTMA-modified loess soil are reduced if two compounds exist simultaneously in the soil, indicating that competitive adsorption between the two hydrophobic aromatic anions occur in soil matrix[14]. Sorption of pentachlorophenol (PCP), a model hydrophobic organic acid, is adequately modeled by accounting for pH- and pKa-dependent chemical speciation and using two organic carbon–normalized sorption coefficients; one each for the neutral and anionic species. Sorption reversibility of PCP by both $CaCl_2$ and

$Ca(H_2PO_4)_2$ solutions was also demonstrated. Results for PCP clearly demonstrate that sorption to anion exchange sites in variable-charge soils should be considered in assessing pesticide mobility and that phosphate fertilizer application may increase the mobility of acidic pesticides[15,16].

### *Enhancing the Sorptive Capabilities of Air and Water Prior to Cleanup*

The behavior of hydrophobic pollutants in moving air or rivers tends to be modified by a reduction in their concentrations because of fairly continuous mixing and dilution. However, contrary to the case of soil, many hydrophobic pollutants tend to accumulate due to the fact that soils act as a sink through adsorption processes, which binds them with varying strengths to the surface of soil colloids. Consequently, adsorption inhibits the leaching of the hydrophobic pollutants down the soil profile to the water table, hence affecting their rate of decomposition. As for relevant environmental factors influencing this adsorption behavior, contents of organic matter and water in soils, soil pH, and air temperature are involved. For example, when soil organic matter or air temperature is reduced and soils are at neutral, runny, or unwatered conditions, the adsorption of the dye on soils are decreased, thereby inhibiting the accumulation of the dye in soil environment[17,18]. Furthermore, the volatilization of hydrophobic pollutants during domestic water usage can lead to significant indoor air concentrations and the subsequent inhalation of these contaminants is an important route of exposure. The magnitude of these exposures is highly dependent on the activities undertaken by the exposed individual, as well as the activities of other occupants of the home[19,20].

## Impact on Environmental Hazards and Risks

### *Effects of Short- and Long-Term Exposures*

The USEPA defines *harm* as anything that adversely affects “the health of living organisms or other interferences with the ecological systems of which they form part and, in the case of man, includes offence caused to any of his senses or harm to his property.” The EPA defines *environment* as consisting of “the air, water, and land; and the medium of air includes the air within buildings and the air within other natural or man-made structures above or below ground.” The environmental hazard and risk to organisms (including man), populations (including man), and ecosystems by hydrophobic pollutants are due to long- and short-term exposures and the inherent potential harm they cause. Exposure depends on both concentration of the hydrophobic pollutants and the period of time. The greater the concentration over a constant time period, the greater the exposure[21,22]. Concentration and period of exposure themselves depend on factors such as properties and behavior of the pollutant, pattern of pollution emission, weather conditions, individual organisms, and population behavior[23,24]. Toxic effects are a subset of harmful effects[25]; therefore, hydrophobic pollutants may exert toxic effects on individual organisms, whole populations, and ecosystems. In marine life, for example, a whole population may be affected[26,27,28]. The effects may be lethal or sublethal as shown in studies by Lloyd[29] and Vanwijnen et al.[30]. Both types of effect are potentially serious. In man, for example, the toxic effects of hydrophobic pollutants may take 2 or more years to be realized, while in some victims, it could take less than 2 years for the toxic effects (especially, respiratory cancer) to be realized[29,30,31]. In this case, the toxic effects often involve specific interactions with key biochemical and physiological systems. The intrinsic potential for any chemical to cause toxic effects is called its *toxicity*. Toxicity of hydrophobic pollutants to man or animals is either by direct contact or by inhalation[2,9,10,30,32,33,34]. The general amount (dose) of hydrophobic pollutant received (i.e., taken into an organism or population) depends on the exposure and a range of other factors: exposed skin surface area (where uptake is via skin), rate of breathing (where uptake is via lungs), temperature, etc.[2,11,30,31,32,35]. Long-term mortality studies of skin and respiratory cancer[29,35,36] among steel and coke plant workers showed that long exposures to hydrophobic pollutants were mostly responsible for a high mortality rate. Soots, tars, and oils are the causes of occupational cancer as a result of long-term exposure[37,38,39]. In this case, exposure is a function of environmental and biological factors. Vanwijnen et al.[30] studied Dutch children who were exposed to PAHs and found that although the children never worked in the factories where the chemicals were released, harmful concentrations were found in their fluid and their health was affected.

### Food Contamination by Hydrophobic Organic Compounds

Several of these organic compounds are known to contaminate foods. For example, PAHs have been detected in both raw and processed foods[40]. The presence of PAHs in nonprocessed foods is associated with environmental pollution from both human and industrial activities, whereas contamination of processed foods can be caused by certain preservation and processing procedures[40]. Both toxicological and epidemiological studies have shown a relation between such compounds and tumor development[41,42,43,44]. The data from Guillen et al.[40] indicate that PAHs must undergo a biotransformation process that causes the formation of biologically active metabolites. In this process, the presence of an enzyme couple that is induced by different xenobiotics is implied, making the toxicity of such compounds hard to predict. Setting a threshold limit below which toxicity could be considered negligible is difficult, therefore, the presence of PAH in foodstuffs should be reduced to as low as possible by controlling environmental contamination and all procedures that could cause PAH contamination during food processing, preserving, and packaging[40,42,44,45]. Several qualitative and semi-quantitative studies have also shown that in aquatic habitats, many organisms, such as fish and shellfish, readily accumulate hydrophobic compounds from the environment and store them in their tissues at relatively high levels. In this context, shellfish have been defined as sensitive indicators of hydrophobic chemicals such as PAHs; contaminant stress is worth noting[44,45,46,47,49]. Lee et al.[2] found that mussels rapidly accumulate PAH, but when transferred to clean water, the mussels are purged of 80–90% of the PAH over a period of 2 weeks.

### Carcinogenicity of Hydrophobic Organic Compounds

Reports issued by the International Agency for Research on Cancer (IARC) describe PAH as an important group of chemical carcinogens or procarcinogens that are widespread as environmental and food contaminants (Table 1)[48,49,50]. Although many studies in the past focused on the carcinogenicity of benzo(a)pyrene, other PAHs and such derivatives as acridines are now well known to have as much biological activity as benzo(a)pyrene. Tremendous work has been done to explain the metabolic pathways in which these compounds play their carcinogenic roles. Historically, on the basis of theoretical developments, a certain area of the molecule of these hydrophobic hydrocarbons, called the K-region, defined as the external corner of a phenanthrenic moiety in a PAH, was related specifically to the carcinogenic effects of such compounds[51]. Tarvis and Hester[50] gave estimated human risk from background exposure to 10 organic pollutants and gave the risk as shown in Table 1. Although this is a risk estimate, corroborated pieces of evidence thus discussed support the fact that the risk of animals and humans developing cancer from exposure to hydrophobic pollutants is relatively higher, especially in a situation where the victims are exposed to a longer period of time[52,53,54,55,56,57,58].

**TABLE 1**
**The Risk of Uptake of 10 Selected Hydrocarbons**

| Route of Uptake and Chemical | RRisk* ($\times 10^{-4}$) |
|---|---|
| Breathing indoor air | |
| Benzene | 1 |
| Trichloroethylene | 0.15 |
| Tetrachloroethylene | 0.12 |
| Carbon tetrachloride | 1.2 |
| Formaldehyde | 0.65 |
| Xylenes | 0.11 |
| Drinking water | |
| Chloroform | 3 |
| Eating food | |
| Dioxins and furans | 2.1 |
| Dieldrin | 0.78 |
| PCBs | 1.1 |

* Risk = probability of developing cancer in lifetime due to exposure route indicated[50].

## *Neurological Effects of Hydrophobic Organic Compounds*

Several exposures to hydrophobic pollutants are known to lead to adverse health manifestations, the effects of which include neurological health problems. Most miners, for example, are in one way or the other exposed to hydrophobic pollutants. Mulloy[57] reported two cases of neurological disease in coal mine preparation plant workers who were environmentally exposed to polymers with acrylamide monomer. In 1992, the two patients were referred to the Division of Occupational and Environmental Health, Department of Family and Community Health, Marshall University School of Medicine, in Huntington, West Virginia for evaluation. The patients had worked in different coal preparation plants in southern West Virginia for over 10 years and had exposure to an acrylamide polymer flocculent contaminated with acrylamide monomer[59,60,61]. Both patients had no instruction on proper rise of, or the dangers of, acrylamide and were not given adequate safety equipment. Patient A developed Parkinsonism and Patient B developed peripheral neuropathies with a neurogenic bladder. These two case reports highlight the need to re-emphasize the basic tenets of occupational health and safety. Many chemicals are being introduced into mining operations without adequate instruction and awareness of the potential toxic exposures. Hence, new diseases previously unreported in the mining industry have now become part of the surveillance system by mine management and labor safety committees in the U.S.[59]. Several other hydrophobic compounds and their derivatives have been implicated in the disruption of the human equilibrium system. The diagnosis of solvent-induced chronic toxic encephalopathy is commonly based on case histories of exposure to solvents, symptoms, and deficits on psychometric tests. However, it has previously been demonstrated that long-term solvent-exposed workers have disturbances of the equilibrium system and incipient, chronic, and nonchronic toxic encephalopathy[60].

## Developmental Effects

Virtually all hydrophobic organic pollutants produce tremendous neurotoxicity against developmental processes. They do so through all or some of their behavioral characteristics. For example, PCBs are a family of chlorinated hydrocarbons that are ubiquitous in the environment. Many of them have very long half-lives in humans and other animals and can be detected in biological tissue in most people in industrialized countries[59,60,61]. Incidents of poisoning from rice oil contaminated with PCBs in Japan and Taiwan revealed hypotonicity and cognitive deficits in infants and children exposed *in utero* in the presence of other signs of toxicity, including low birth weight, abnormal pigmentation, and swollen gums and eyelids. Several other research studies[59,60,61,62] also reported decreased IQ and reading ability when children were 11 years old, decreased reflexes and retarded psychomotor development during infancy, and changes in activity and cognitive function as a result of developmental exposure to PCBs.

## CONCLUSIONS

The influence of hydrophobic pollutant behavior on environmental hazards and risks has been discussed. Although many studies have been done on the persistence, bioaccumulation potential, toxicity, environmental mobility, and reactivity of hydrophobic pollutants, a lot more qualitative and semi-quantitative approaches should be adopted to assess their potential to cause harm to organisms including man and ecosystems. For example, more feasible monitoring, estimating, and modeling mechanisms should be devised to enable prediction of environmental exposure (distribution or pathway fate) and assessment of harmful effects with reference to specified target(s). At present, the probability of specific harm occurring as a result of a particular situation is still debatable since most of the evidence available is statistically and quantitatively based. Risk tends to be used in more specific and well-defined situations. Risk estimation or assessment should be carried out to monitor and control the sources of activities, release, contamination, and pollution of the environment by hydrophobic chemicals. A stricter environmental regulatory mechanism should be devised to oversee manufacturing, application, leakage, and waste disposal procedures and practices.

## BIOSKETCHES

**Ijeoma Kanu, MSc, PhD** (Candidate) is a lecturer in the Department of Microbiology, Abia State University and a PhD Research Student in environmental microbiology at the Michael Okpara University of Agriculture, Umudike, Umuahia, Abia State Nigeria. E-mail: ijay2001kay@yahoo.com

**Ebere C. Anyanwu, PhD** is a Research Scientist at the Medical Center for Immune and Toxic Disorders and also Adjunct Professor of Anatomy and Physiology at the North Harris and Montgomery College District, Houston, TX. E-mail: ebereanyanwu@msn.com
